# Hours Lost to Planned and Unplanned Dental Visits Among US Adults

**DOI:** 10.5888/pcd15.170225

**Published:** 2018-01-11

**Authors:** Uma Kelekar, Shillpa Naavaal

**Affiliations:** 1 Department of Health Care Management and Legal Studies, Marymount University, Arlington, Virginia; 2 Department of Oral Health Promotion and Community Outreach, Philips Institute for Oral Health Research, Virginia Commonwealth University, Richmond, Virginia

## Abstract

**Introduction:**

Poor oral health is associated with lost hours at work or school, which may affect a person’s productivity. The objective of our study was to estimate work or school hours lost to dental visits among adults aged 18 and older by the types of visits (emergency or unplanned; routine, planned, or orthodontic; or cosmetic) and to determine the factors associated with hours lost.

**Methods:**

We used the most recent Oral Health Supplement data, from the 2008 National Health Interview Survey (NHIS), to estimate the total hours lost at work or school for dental visits among adults in the United States. The associations of the hours lost in unplanned and planned dental visits with socioeconomic characteristics, oral health status, and affordability were calculated. We used χ^2^ tests and logistic regression to determine associations at *P* < .05.

**Results:**

An average of 320.8 million work or school hours were lost annually for dental care in the United States, of which 92.4 million hours were for emergency (unplanned) care (0.99 h/adult), 159.8 million for routine (planned) care or orthodontic care (1.71 h/adult), and 68.6 million for cosmetic care (0.73 h/adult). Adults with poor oral health were more likely to lose one or more hours in unplanned dental visits (OR = 5.60; 95% confidence interval [CI], 3.25–9.63) than those who reported very good oral health. Not being able to afford dental care was positively associated with more work hours lost in unplanned care (odds ratio [OR] = 2.56; 95% CI, 1.76–3.73). Compared with Hispanic adults, non-Hispanic white adults (OR = 2.09; 95% CI, 1.40–3.11) and non-Hispanic Asian adults and adults of other races/ethnicities (OR =1.91; 95% CI, 1.06–3.47) were more likely to lose any hours for planned care. Consistently, those with more than a high school education were more likely to lose any hours in planned care (OR = 1.39; 95% CI, 1.06–1.83) than those with a high school education or less.

**Conclusions:**

Dental problems result in hours lost from work and may adversely affect a person’s productivity. There is disparity in lost hours at work by race/ethnicity and dental care affordability.

## Introduction

The most common reason for adults to forgo dental care is cost (1). Unmet oral health needs not only incur costs to treat associated diseases but may also affect a person’s productivity and income. Productivity losses may be in the form of work hours lost for dental visits, emergency department visits, and potential life years lost to premature death. Among children and adults, the effects of oral disease may include school days lost, challenges in learning, social stigma, or impaired nutrition and health (2).

One study that used data from the 1989 National Health Interview Survey (NHIS) measured time missed from work and school because of dental problems or dental visits among adults (3). That study found that employed people missed 164 million work hours (1.48 h/person) for dental visits. A longitudinal study found that 26.4% of working adults in their sample reported an episode of dental-related work loss with a mean loss of 1.26 hours per person per year (4). The objective of our study was to estimate work or school hours (hereinafter hours) lost for dental visits by type of visits. We categorized visits as unplanned (emergency care), planned (routine and orthodontic care), and cosmetic. We also examined factors associated with time lost.

## Methods

We used secondary data from the 2008 National Health Interview Survey (NHIS) core module and oral health supplement for adults aged 18 or older (5). NHIS, conducted by the Centers for Disease Control and Prevention, is a principal source of data on the health behaviors of the civilian, noninstitutionalized household population of the United States. Since 1957, along with the core survey, NHIS included optional oral health supplements in years 1989, 1999, and 2008. For this study, we merged 2008 NHIS family, person, and sample adult files.

### Outcome and explanatory variables

To determine hours lost for unplanned, planned, and cosmetic dental care, the survey asked the following 3 questions: “Please tell me how many hours of work or school were missed in the past 6 months for:

- emergency dental care where you saw the dentist within 24 hours or as soon as was possible,
- planned routine dental or orthodontic care,
- or [planned] tooth whitening or other cosmetic procedures.”

These questions were asked only of adults who visited a dentist in the past 6 months. Therefore, of the total 21,781 participants, 8,713 were eligible to answer and made up our study sample.

Possible responses for hours lost in each type of dental care were none to less than 1 hour, 1 hour to less than 3 hours, 3 hours to less than 5 hours, 5 hours to less than 7 hours, and more than 7 hours; “did not work or go to or school”; and “did not have this type of dental care.” Responses of “did not know,” “refused to answer,” or “could not be ascertained” were set to missing because of our small sample size. The outcome variables were the proportions of people who lost hours. To obtain the average number of hours lost, we aggregated the midpoint hours from individual responses. For those with a response of 7 or more hours, we assumed 7 hours lost. To be consistent with a previous study that reported annual work hours lost (3), we doubled our estimate of total hours to obtain an annual estimate.

For the bivariate and the multivariate analyses, our primary variables of interest were hours lost because of unplanned or planned dental care. Other variables were age in years (18–24, 25–44, 45–64, and ≥65); sex; education level (high school or less education, more than high school education); race/ethnicity (Hispanic, non-Hispanic white, non-Hispanic black, non-Hispanic Asian, or other); annual family income (<$35,000, $35,000–$74,999, $75,000–$99,000, or ≥$100,000); oral health condition or status (very good, good, fair, poor); and dental care affordability (yes/no).

### Statistical analysis

Data management and analyses were conducted by using STATA, version 13 (STATA Corp) to account for survey weighting and to adjust the variance for the multistage, clustered survey design. Sampling weights provided by the NHIS data set were used to generalize the estimates to the US civilian, noninstitutionalized adult population.

χ^2 ^ tests and multivariate regressions were conducted to determine the factors associated with loss of hours for both outcomes, hours lost for unplanned and planned dental care. We wanted to know whether the factors that affect losing any hours versus losing none and factors that affect losing less than one hour versus more than one hour were different. Hence, for each of the outcome variables, we created 2 sets of logistic regression models. We used 4 logistic regression models to estimate the factors associated with hours missed for unplanned and planned dental care. Models 1 and 3 compared those who lost any hours with those who did not lose any hours for unplanned and planned care, respectively. Models 2 and 4 compared those who lost less than 1 work hour to those who lost 1 hour or more for unplanned and planned dental care, respectively. All models excluded adults who did not work or go to school. Estimates with more than 30% relative standard error were considered statistically unstable. *P* < .05 was considered significant.

## Results

Among the 8,713 adults who visited a dentist in the past 6 months and were asked about the number of hours lost for dental care, 55% were women, 83% were aged 18 to 64 years, and 9% were of Hispanic origin. Almost 80% had more than a high school education, and 49% had annual incomes below $75,000. Most (92%) could afford dental care, and 83% reported their oral health status as very good or good.

Among adults who visited a dentist in the past 6 months, 320.8 million hours were lost because of dental visits that were unplanned, planned, or cosmetic. A total of 92.4 million hours (0.99 h/adult) were lost for unplanned visits (29% of total time lost); 159.8 million work hours (1.71 h/adult) were lost for planned visits (50% of total time lost); and 68.6 million work hours (0.73 h/adult) were lost for cosmetic dental visits (21% of total time lost). For every 1,000 adults, 986 hours were lost for unplanned care, 1,706 hours for planned care, and 732 hours for cosmetic care. In this article, we focused our bivariate and multivariate analyses on unplanned and planned dental care visit data.

**Emergency or unplanned dental care**. A total 8,635 adults had a nonmissing response to the question about unplanned dental care in the past 6 months. Among these, 63.1% reported losing less than 1 hour, 4.4% reported losing 1 or more hours, 20.8% did not need this type of care (Table 1), and those remaining did not work or go to school. Overall, 67.5% of the population lost any hours in seeking unplanned dental care.

**Table 1:** Demographic Characteristics of Working Adults Who Lost Work Hours for Dental Care in Past 6 Months, 2008 National Health Interview Survey (NHIS), Oral Health Supplement^a^

Characteristic	Hours Lost, Emergency or Unplanned Care, % (SE)			*P* Value^b^	Hours Lost, Routine, Planned, or Orthodontic Care, % (SE)			*P* Value^b^
	<1 h	≥1 h	Did Not Need This Type of Care		<1 h	≥1 h	Did Not Need This Type of Care	
**Total**	63.1 (0.9)	4.4 (0.2)	20.8 (0.8)	—	63.9 (0.8)	17.1 (0.6)	5.8 (0.4)	—
**Sex**								
Male	63.1 (1.2)	4.7 (0.4)	22.6 (1.0)	<.001	63.1 (1.1)	19.3 (0.8)	7.0 (0.6)	<.001
Female	63.2 (1.0)	4.2 (0.3)	19.4 (0.9)		64.5 (0.9)	15.4 (0.7)	4.9 (0.4)	
**Age, y**								
18–24	64.4 (2.9)	5.5 (1.0)	25.6 (2.8)	<.001	71.0 (2.2)	18.0 (1.9)	5.4 (1.1)	<.001
25–44	67.0 (1.1)	6.5 (0.5)	22.1 (1.0)		68.4 (1.2)	20.3 (0.9)	6.0 (0.6)	
45–64	66.4 (1.2)	3.9 (0.4)	20.5 (1.1)		63.8 (1.1)	19.6 (0.8)	6.4 (0.6)	
≥65	47.4 (1.5)	0.7 (0.2)	16.2 (1.0)		50.7 (1.5)	4.8 (0.6)	4.4 (0.6)	
**Race/ethnicity**								
Hispanic	62.2 (2.2)	6.3 (1.2)	21.3 (1.7)	.02	64.1 (2.2)	14.3 (1.3)	10.4 (1.5)	<.001
Non-Hispanic white	62.6 (1.1)	4.2 (0.3)	20.9 (0.9)		63.4 (0.9)	17.7 (0.7)	5.1 (0.4)	
Non-Hispanic black	65.9 (2.1)	5.6 (0.8)	19.1 (1.7)		65.3 (2.2)	16.3 (1.5)	7.9 (1.4)	
Non-Hispanic Asian, other race/ethnicity	67.4 (2.4)	1.9 (0.5)	21.0 (2.2)		68.1 (2.1)	15.5 (1.6)	5.4 (1.1)	
**Education^b^**								
High school education or less	58.4 (1.4)	4.8 (0.6)	17.8 (1.1)	<.001	58.7 (1.4)	12.4 (0.8)	7.9 (0.8)	<.001
More than high school education	64.4 (1.1)	4.3 (0.3)	21.6 (0.9)		65.2 (0.8)	18.4 (0.6)	5.3 (0.4)	
**Annual household income^c^, $**								
<35,000	55.2 (1.7)	4.7 (0.5)	19.2 (1.6)	<.001	58.7 (1.6)	11.7 (0.9)	6.9 (0.8)	<.001
35,000–74,999	61.5 (1.2)	4.6 (0.5)	20.7 (1.1)		63.6 (1.2)	15.5 (0.9)	6.0 (0.6)	
75,000–99,000	66.8 (19)	3.8 (0.7)	23.3 (1.7)		70.7 (1.4)	16.8 (1.2)	4.9 (0.8)	
≥100,000	68.4 (1.6)	5.0 (0.6)	20.9 (1.3)		65.0 (1.4)	23.3 (1.1)	5.0 (0.6)	
**Oral health status^c^**								
Very good	67.3 (1.3)	2.2 (0.3)	21.9 (1.1)	<.001	67.9 (1.0)	17.0 (0.8)	5.1 (0.5)	<.001
Good	61.7 (1.2)	4.5 (0.4)	21.6 (1.0)		62.7 (1.0)	17.0 (0.7)	6.6 (0.6)	
Fair	58.5 (1.8)	8.8 (0.9)	16.2 (1.3)		58.3(1.6)	18.2 (1.3)	5.6 (0.8)	
Poor	47.2 (3.5)	13.4 (2.5)	15.9 (2.5)		50.6 (3.3)	17.7 (2.5)	6.8 (1.4)	
**Affordability of dental care**								
Cannot afford	59.3 (2.3)	14.4 (1.8)	14.6 (1.5)	<.001	61.4 (2.2)	21.7 (1.9)	4.8 (0.9)	.03
Can afford	63.4 (0.9)	3.6 (0.2)	21.3 (0.8)		64.1 (0.7)	16.8 (0.6)	5.9 (0.4)	

Abbreviation: SE, standard error.

a Sample consisted of 8,713 adults who indicated they had visited a dentist in the past 6 months. Sampling weights provided by the NHIS data set were used to generalize the estimates to the US civilian, noninstitutionalized adult population.

b χ^2^ tests were used to test for associations.

c Less than 1% of sample had missing values for education and oral health; 8.3% of the sample had missing values for income.

Adults aged 25 to 44 years (6.5%) and Hispanic adults (6.3%) lost 1 hour or more for unplanned care. A larger proportion of those with more than a high school education (68.7%) and those with annual incomes above $100,000 (73.4%) lost any hours seeking unplanned dental care compared with those with less than a high school education (63.2%) or those with annual incomes below $35,000 (59.9%). Approximately 13% of adults with poor oral health and 14% of adults who could not afford dental care lost an hour or more for unplanned dental care compared with those with very good oral health (2.2%) or those who could afford dental care (3.6%) (Table 1).

**Routine, planned, or orthodontic dental care**. A total of 8,630 adults had a nonmissing response to the question about planned dental care. Among these, 63.9% reported losing less than 1 hour in the past 6 months, 17.1% reported losing 1 or more hours, 5.8% did not need this type of care (Table 1), and the remainder did not work or go to school. Overall, 81% of the population lost any hours in seeking planned dental care.

Approximately 19% of men and nearly 20% of those aged 25 to 44 and 45 to 64 lost 1 hour or more compared with 15.4% of women and 4.8% of those aged 65 or older. Among adults with annual incomes less than $35,000, 11.7% lost 1 hour or more, and 12.4% of those with high school education or less lost 1 hour or more compared with 23.3% of adults with annual incomes over $100,000 and 18.4% of those with more than high school education Among those who reported very good oral health, 85% lost work hours compared with 68% of those who reported poor oral health. Furthermore, 21.7% of those who could not afford dental care lost an hour or more in seeking planned care compared with 16.8% of those who could afford dental care (Table 1).

### Regression results

**Unplanned dental care**. When comparing those who lost hours with those who did not, women had greater odds of losing any hours in seeking unplanned care (OR = 1.21; 95% CI, 1.07–1.36) compared with men (Table 2, Model 1). Adults aged 45 to 65 (OR = 0.62; 95% CI, 0.39–0.98) and over 65 (OR = 0.19; 95% CI, 0.09–0.39) had lower odds of losing 1 or more hours for unplanned dental care than those aged 18 to 24 years (Table 2, Model 2).

**Table 2:** Multivariate Logistic Regression Analysis of Odds of Losing Work Time for Dental Care in the Past 6 Months Among Working Adults Aged18 or Older, 2008 National Health Interview Survey (NHIS), Oral Health Supplement^a^

Variable	Odds Ratio (95% Confidence Interval)			
	Emergency or Unplanned Care		Routine, Planned, or Orthodontic Care	
	Model 1 (n = 6,921)	Model 2 (n = 5,336)	Model 3 (n = 6,799)	Model 4 (n = 6,336)
**Sex**				
Male	1 [Reference]			
Female	1.21 (1.07–1.36)	0.92 (0.71–1.21)	1.51 (1.20–1.90)	0.79 (0.68–0.92)
**Age, y**				
18–24	1 [Reference]			
25–44	1.15 (0.86–1.53)	0.97 (0.61–1.53)	0.81 (0.49–1.34)	1.08 (0.80–1.46)
45–64	1.19 (0.87–1.61)	0.62 (0.39–0.98)	0.69 (0.42–1.15)	1.09 (0.81–1.47)
≥65	1.09 (0.79–1.49)	0.19 (0.09–0.39)	0.78 (0.50–1.22)	0.35 (0.24–0.50)
**Race/ethnicity**				
Hispanic	1 [Reference]			
Non-Hispanic white	1.08 (0.86–1.37)	0.87 (0.55–1.39)	2.09 (1.40–3.11)	1.24 (0.95–1.61)
Non-Hispanic black	1.26 (0.91–1.73)	0.91 (0.54–1.54)	1.56 (0.92–2.63)	1.08 (0.77–1.51)
Non-Hispanic Asian and other race/ethnicity	1.04 (0.74–1.48)	0.38 (0.19–0.75)	1.91 (1.06–3.47)	0.99 (0.69–1.40)
**Education**				
High school education or less	1 [Reference]			
More than high school education	0.82 (0.68–1.00)	0.92 (0.65–1.29)	1.39 (1.06–1.83)	1.14 (0.92–1.42)
**Annual household income, $**				
<35,000	1 [Reference]			
35,000–74,999	1.06 (0.86–1.30)	1.04 (0.71–1.53)	1.24 (0.91–1.69)	1.16 (0.92–1.46)
75,000–99,000	1.06 (0.81–1.38)	0.95 (0.57–1.60)	1.60 (1.04–2.44)	1.08 (0.83–1.40)
≥100,000	1.26 (0.95–1.67)	1.49 (0.95–2.32)	1.50 (1.00–2.25)	1.63 (1.26–2.10)
**Oral health status**				
Very good	1 [Reference]			
Good	0.97 (0.83–1.14)	2.18 (1.51–3.16)	0.79 (0.61–1.03)	1.15 (0.99 –1.33)
Fair	1.15 (0.92–1.44)	4.15 (2.70–6.39)	0.93 (0.65–1.33)	1.37 (1.09–1.73)
Poor	0.97 (0.65–1.46)	5.60 (3.25–9.63)	0.61 (0.38–0.97)	1.39 (0.93–2.06)
**Affordability of dental care**				
Can afford	1 [Reference]			
Cannot afford	1.64 (1.24–2.15)	2.56 (1.76–3.73)	1.57 (1.01–2.43)	1.32 (1.01–1.72)

a Sample consisted of 8,713 adults who indicated they had visited a dentist in the past 6 months. Sampling weights provided by the NHIS data set were used to generalize the estimates to the US civilian, noninstitutionalized adult population. Models 1 and 3 compared those who lost time with those who did not lose any time for dental care. Models 2 and 4 compared those who lost less than 1 work hour with those who lost 1 or more hours.

In both models, affordability was a strong predictor of hours lost in seeking unplanned care. In Model 1, those who could not afford dental care had greater odds of losing hours (OR = 1.64; 95% CI, 1.24–2.15) than those who could afford dental care. In Model 2, those who could not afford dental care (OR = 2.56; 95% CI, 1.76–3.73) or those who reported good (OR = 2.18; 95% CI, 1.51–3.16), fair (OR = 4.15; 95% CI, 2.69–6.39) or poor oral health (OR = 5.60; 95% CI, 3.25–9.63) had greater odds of losing 1 or more hours for unplanned care than those who could afford care or had very good oral health.

**Planned dental care**. Sex, age, race/ethnicity, education, income, oral health, and dental care affordability were predictors of hours lost for planned care. In Model 3, women were more likely to lose hours than men (OR = 1.51; 95% CI, 1.20–1.90); however, when women sought care, they were less likely to lose more hours than men (OR = 0.79; 95% CI, 0.68–0.92) (Table 2, Model 4).

Compared with Hispanic adults, non-Hispanic white adults (OR = 2.09; 95% CI, 1.40–3.11), non-Hispanic Asian and other race/ethnicities (OR = 1.91; 95% CI, 1.06–3.47) were more likely to lose any hours for planned care (Table 2, Model 3). Consistently, those with more than a high school education were more likely to lose any hours (OR = 1.39; 95% CI, 1.06–1.83) than those with high school or less.

In Model 4, those aged 65 or older were less likely to lose more hours than those aged 18 to 24 (OR = 0.35; 95% CI, 0.24–0.50). In addition, those with incomes more than $100,000 were more likely to lose more hours in seeking planned care compared to those with incomes less than $35,000 (OR = 1.63; 95% CI, 1.26–2.10). Furthermore, those in fair health (OR = 1.37; 95% CI, 1.09–1.73) or those who could not afford care were more likely (OR = 1.32; 95% CI, 1.01–1.72) to lose more hours in seeking planned care than those with very good health or those who could afford care.

## Discussion

This study provides data on the number of work hours missed in a year for unplanned dental care (92.4 million hours), planned dental care (159.8 million hours), and cosmetic care (68.6 million hours) by using the most recent NHIS data (5). Disparities by socioeconomic factors exist in work hours lost among adults. To the best of our knowledge, ours is the first study since 1992 (3) to estimate hours lost for dental visits. In addition to providing data on total hours lost, we provided a breakdown of hours lost by type of dental care and also examined factors associated with hours lost for unplanned and planned dental care.

Consistent with previous studies (4,6), women were more likely to lose hours to planned care than men, while those aged 65 or more were less likely to lose more hours in planned and unplanned care than those aged 18 to 24. A significant portion of the elderly are toothless or have few teeth (7), which could account for their losing less time to dental care. We found that although women were more likely to seek planned dental care, they were less likely to lose 1 hour or more for those visits than men. One explanation could be that women generally use more health services than men (8). However, because they seek regular routine oral care, they may be less likely to need extensive care and therefore less time off from work or school.

Consistent with previous studies (4,6), we found that adults with more than a high school education or more than $75,000 in annual income to be more likely to lose hours in planned care than those with less education or lower incomes. Furthermore, the Hispanic adult population was less likely to lose hours in planned care than its non-Hispanic white or Asian and other counterparts.

The reasons for disparities in oral health are complex. Low-income people or those of racial/ethnic minority groups may not have regular access to dental care, or factors such as high cost, lack of transportation, lack of access to a provider, or lack of flexibility to take time off from work may affect their ability to use dental care (9). Moreover, adults from low socioeconomic groups may have more limited resources to plan ahead than adults from upper socioeconomic groups (3).

Oral health was associated with more time lost for unplanned care. Although we were not able to observe the problems that led to dental visits, the significant number of hours lost in unplanned care may suggest a high burden of dental disease among these adults. Moreover, the strong association of lack of affordability with the loss of hours for unplanned dental care indicates how the inability to pay may prompt some users to seek care only when needed. Perhaps the lack of regular preventive dental care delays diagnosis of oral health problems that in turn causes adults to lose time in seeking unplanned care.

The hospital emergency department (ED) may be one of the providers of unplanned or urgent care. Although less than 1% (0.69%) of total dental visits in 2013 were in the ED (10), most of those patients were of working age (18–44, according to the 2009 Nationwide Emergency Department Sample data) (11). Studies have shown that dental treatment in a hospital ED is not cost-effective. Moreover, treatment in the ED is focused on pain alleviation rather than dental procedures (12). From a policy perspective, emergency dental visits to the ED point to the need for making dental care affordable and accessible to all. A few other country-level studies have estimated the work days lost because of dental problems. A Canadian study estimated a loss of over 40 million hours (3.5 h/person) from dental problems (6). Another study of Australian workers estimated an average loss of 1.56 hours per worker from dental problems (13). We found that, on an average, a US adult lost approximately 3.5 hours annually to dental visits. Although this may not seem like a significant loss at an individual level, it may be significant at a societal level.

Our results do not compare directly with those of Gift et al (3). However, it is noteworthy that we found that a significant number of work hours were lost to dental care. In the United States, most dental expenses (90%) are paid for privately, mainly out-of-pocket or by insurance (14). The remaining 10% is funded by government, mostly through Medicaid. Our findings indicated that although 81% of the population that visited a dentist did so for planned care, 68% did so for unplanned care. At a policy level, the lack of affordable dental care among adults may partially explain these findings.

Our study had limitations. First, we estimated hours lost to unplanned visits; however, measuring hours lost because of dental pain or other symptoms prior to that visit was beyond the scope of our study, probably causing an underestimate of the overall loss of hours. Second, the total number of hours missed were self-reported by adults, which may be subject to recall bias or social desirability bias. Third, because the question was asked only of those who visited the dentist in the past 6 months, total hours might have been underestimated. Fourth, because of the nature of the question, hours lost in planned dental care include routine and orthodontic care, and we were unable to differentiate the proportions of each service. Finally, because the survey was cross-sectional, it was not possible to examine whether and why adults used follow-up care, which might have provided additional insights about the reasons for dental visits.

Potential employers might have significant interest in a study that examines how dental problems relate to work hours lost. As a strategy to avoid work hours lost from unplanned dental care, employers and insurers could consider wellness programs that integrate preventive dental care. A few studies investigated the effect of dental pain on absenteeism in the Brazilian workplace (15,16). In addition to exploring the association between pain and absenteeism among adults, future research could focus on examining work time lost across occupational groups and the associated productivity losses measured in terms of lost wages. Also, a longitudinal analysis may provide more insight into how people use follow-up care after an unplanned visit to the dentist, if at all.

Significant time is lost from work or school by adults for unplanned dental care in United States. Findings from this study should be used to reduce the burden of unplanned dental visits and improve use of routine dental care among the adult working population.
